# Cascade-responsive nanobomb with domino effect for anti-tumor synergistic therapies

**DOI:** 10.1093/nsr/nwab139

**Published:** 2021-08-09

**Authors:** Yang Liu, Yinghui Wang, Shuyan Song, Hongjie Zhang

**Affiliations:** State Key Laboratory of Rare Earth Resource Utilization, Changchun Institute of Applied Chemistry, Chinese Academy of Sciences, Changchun 130022, China; School of Applied Chemistry and Engineering, University of Science and Technology of China, Hefei 230026, China; State Key Laboratory of Rare Earth Resource Utilization, Changchun Institute of Applied Chemistry, Chinese Academy of Sciences, Changchun 130022, China; State Key Laboratory of Rare Earth Resource Utilization, Changchun Institute of Applied Chemistry, Chinese Academy of Sciences, Changchun 130022, China; School of Applied Chemistry and Engineering, University of Science and Technology of China, Hefei 230026, China; State Key Laboratory of Rare Earth Resource Utilization, Changchun Institute of Applied Chemistry, Chinese Academy of Sciences, Changchun 130022, China; School of Applied Chemistry and Engineering, University of Science and Technology of China, Hefei 230026, China; Department of Chemistry, Tsinghua University, Beijing 100084, China

**Keywords:** reactive oxygen species, immunotherapy, chemiexcited photodynamic therapy, ion interference therapy, biodegradable

## Abstract

The development of reactive oxygen species (ROS) generation agents that can selectively produce sufficient ROS at the tumor site without external energy stimulation is of great significance for the further clinical application of ROS-based therapies. Herein, we designed a cascade-responsive ROS nanobomb (ZnO_2_@Ce6/CaP@CPPO/BSA, designated as Z@Ce6/CaP@CB) with domino effect and without external stimulation for the specific generation of multiple powerful ROS storms at the tumor site. The calcium phosphate shell and ZnO_2_ core gradually degrade and release Ca^2+^, Zn^2+^ and hydrogen peroxide (H_2_O_2_) under acid stimulation. On the one hand, Zn^2+^ can enhance the generation of endogenous superoxide anions (·O_2_^–^) and H_2_O_2_ through the inhibition of the mitochondrial electron transport chain. On the other hand, the generation of large amounts of exogenous H_2_O_2_ can cause oxidative damage to tumor cells and further activate bis[2,4,5-trichloro-6-(pentyloxycarbonyl)phenyl] oxalate (CPPO)-mediated chemiexcited photodynamic therapy. In addition, the oxidative stress caused by the generated ROS can lead to the uncontrolled accumulation of Ca^2+^ in cells and further result in Ca^2+^ overload-induced cell death. Therefore, the introduction of Z@Ce6/CaP@CB nanobombs triggered the ‘domino effect’ that caused multiple heavy ROS storms and Ca^2+^ overload in tumors and effectively activated anti-tumor immune response.

## Teaser

Although more attention has been given to cancer therapy based on reactive oxygen species (ROS) in recent years, the ROS-generation efficiency of the drug has received multiple restrictions of exogenous and endogenous stimulation. How to selectively generate sufficient ROS without external energy stimulation under mild *in vivo* conditions remains a huge challenge in the field of cancer therapy.

## INTRODUCTION

Reactive oxygen species (ROS) can act as signal carriers during the evolution of malignant tumors [1]. At the appropriate concentration, ROS (such as hydrogen peroxide (H_2_O_2_), superoxide anions (·O_2_^–^) and hydroxyl radicals (·OH)) mediate signal transduction and cell growth [2]. However, ROS are a double-edged sword, as excessive ROS can oxidize proteins, damage the DNA structure and induce cell apoptosis [3–6]. Moreover, ROS can induce inflammation at the tumor site, which further improves tumor immunogenicity [7]. Therefore, increasing the content of ROS in tumor sites has become an effective method for cancer therapy. At present, the ways to generate ROS through external stimulations, such as photodynamic reaction (photodynamic therapy (PDT)) [8,9], sonodynamic reaction (sonodynamic therapy (SDT)) [10,11] and radiation sensitization (radiotherapy (RT)) [12,13], are greatly limited by the penetration depth of the laser, irradiation range of external excitation and safety concerns of the radiation.

In response to these problems, chemodynamic therapy (CDT) has been developed, which has received widespread attention [14–16]. CDT uses excess H_2_O_2_ in the tumor microenvironment (TME) without external energy stimulation to generate ROS through the Fenton reaction. However, the current therapeutic effect of CDT is not satisfactory, because the initiation of an efficient Fenton reaction requires harsh acidic conditions (pH 3–4) and excess H_2_O_2_ [17]. In addition to exogenous ROS production strategies, increasing the generation of endogenous ROS to inhibit tumor growth is another promising approach. It is well known that inhibiting the mitochondrial electron transport chain (ETC) can enhance the generation of ·O_2_^–^ and H_2_O_2_ [18]. A variety of ETC inhibitors have been reported to effectively kill tumor cells [19]. However, treating cancer only by increasing endogenous ROS is unsatisfactory, as it is difficult to effectively inhibit tumor growth with a low amount of produced endogenous ROS. Therefore, developing strategies for the selective generation of sufficient ROS without external energy stimulation under mild *in vivo* conditions remains a huge challenge in the field of cancer therapy.

**Scheme 1.**
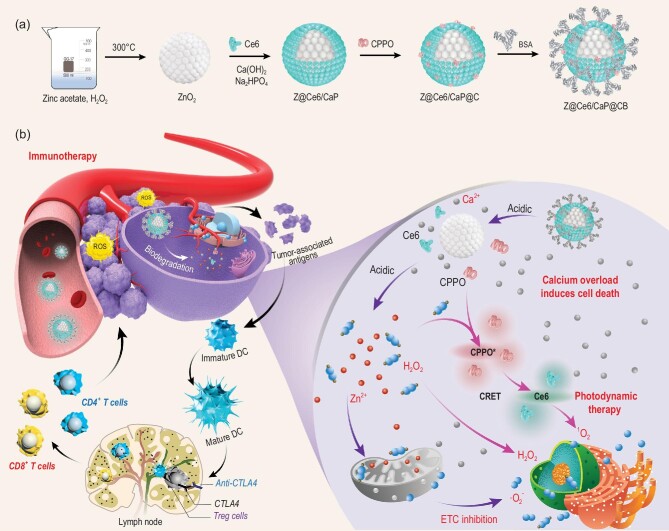
(a) Synthesis process of Z@Ce6/CaP@CB. (b) Schematic illustration of the mechanism of Z@Ce6/CaP@CB-based synergistic therapy. Z@Ce6/CaP@CB nanobombs can trigger multiple ROS storms (H_2_O_2_, ^1^O_2_ and ·O_2_^–^) and Ca^2+^ overload-induced cell death through a cascade reaction without external energy activation and effectively activate the systemic immune response while inhibiting the growth of primary tumors.

Herein, we designed a cascade-responsive ROS-generation device with domino effect and without external stimulation for the specific generation of multiple severe ROS storms at the tumor site. As shown in Scheme 1, zinc peroxide nanospheres (ZnO_2_) were prepared, and were coated with a calcium phosphate (CaP) shell loaded with the photosensitizer chlorin e6 (Ce6) through an *in situ* template-assisted strategy (designated as Z@Ce6/CaP). Then, the surface of Z@Ce6/CaP (designated as Z@Ce6/CaP@CB) was further modified with bis[2,4,5-trichloro-6-(pentyloxycarbonyl)phenyl] oxalate (CPPO) and bovine serum albumin (BSA). Z@Ce6/CaP@CB gradually degraded and produced a variety of ROS through a cascade reaction under acid stimulation. The CaP shell on the surface will be degraded to release Ce6, Ca^2+^ and CPPO. ZnO_2_ can be further degraded to produce Zn^2+^ and H_2_O_2_ under acid stimulation. On the one hand, Zn^2+^ can enhance the generation of ·O_2_^–^ and H_2_O_2_ through the inhibition of the mitochondrial ETC, achieving a rapid increase in endogenous ROS. On the other hand, the large amount of H_2_O_2_ produced will quickly increase the ROS threshold at the tumor site, causing oxidative damage to tumor cells, but can also be used as another stimulus to activate CPPO-mediated chemiexcited PDT. The chemical energy generated by H_2_O_2_-triggered CPPO activation can further stimulate Ce6 to produce ^1^O_2_ through chemiluminescence resonance energy transfer (CRET) without excitation by any external light source [20,21]. As the second messenger of intracellular signal transmission, Ca^2+^ plays a vital role in the process of regulating various physiological functions. Under normal circumstances, cells have extremely strict regulatory mechanisms for Ca^2+^. However, under oxidative stress, the ability of cells to regulate Ca^2+^ decreases gradually, leading to intracellular calcium overload. Therefore, the oxidative stress caused by the generated ROS (H_2_O_2_, ^1^O_2_ and ·O_2_^–^) can cause an uncontrolled accumulation of Ca^2+^ in cells. Obstruction of the accurate transmission of Ca signals will further induce cell death [22,23]. Therefore, the simple introduction of the prepared Z@Ce6/CaP@CB nanobomb into the tumor would cause a ‘domino effect’, which could trigger the production of multiple ROS storms and Ca^2+^ overload, as well as effectively activate the systemic immune response while inhibiting the growth of primary tumors. Moreover, tumor metastasis can be effectively prevented by adjuvant treatment with anti-CTLA4 checkpoint blockers.

## RESULTS AND DISCUSSION

**Figure 1.**
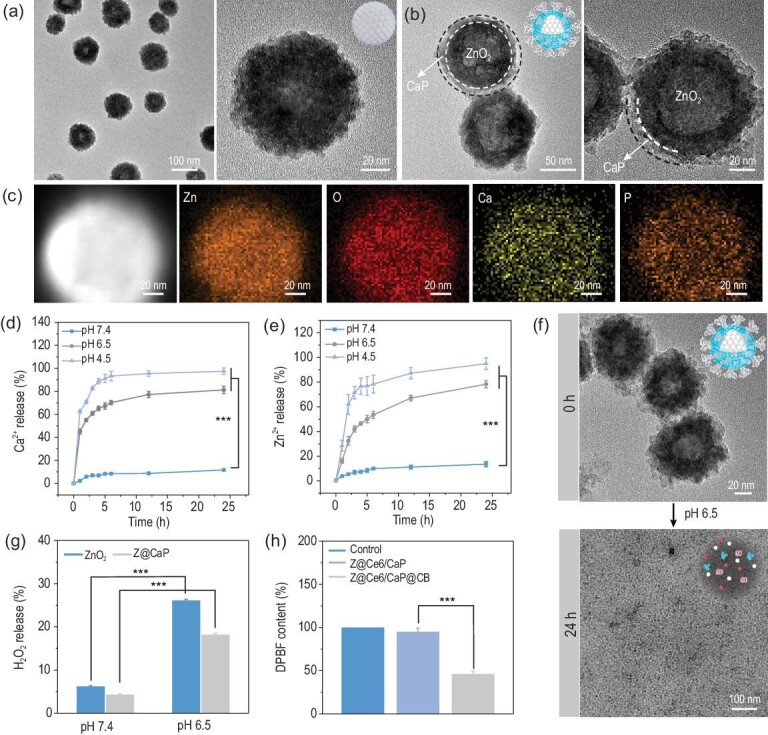
TEM images of (a) ZnO_2_ and (b) Z@Ce6/CaP@CB. (c) High-angle annular dark-field (HAADF)-scanning transmission electron microscopy (STEM) image and elemental mapping of Zn, O, Ca and P of Z@Ce6/CaP. (d) Ca^2+^ and (e) Zn^2+^ release in PBS solutions (pH = 4.5, 6.5 and 7.4) at different time points (1, 2, 3, 4, 5, 6, 12 and 24 h). (f) TEM images of Z@Ce6/CaP@CB in PBS solution (pH = 6.5) at 0 and 24 h. (g) H_2_O_2_ release in PBS solutions (pH = 6.5 and 7.4) at 2 h. (h) Depletion of DPBF due to CPPO-mediated chemiexcited PDT: (i) Control, (ii) Z@Ce6/CaP and (iii) Z@Ce6/CaP@CB. ^***^p < 0.001 by Student's two-tailed t test.

ZnO_2_ nanospheres were prepared according to a previously reported method [24]. An aqueous solution of zinc acetate was mixed with H_2_O_2_ and immediately heated in a superheated plate at a temperature of 300°C. Transmission electron microscope (TEM) images of ZnO_2_ showed a spherical structure with a uniform size of ∼80 nm (Fig. 1a and Fig. S1, Supplementary Data). Then, ZnO_2_ was coated with a CaP protective layer to form Z@CaP through an *in situ* template-assisted strategy [25]. We directly added Ce6 during the CaP coating process to achieve efficient loading and packaging of the photosensitizer (Z@Ce6/CaP). Scanning electron microscopy (SEM) and TEM images (Fig. 1b and Figs S2 and S3) showed a shell with a thickness of ∼10 nm around ZnO_2_ after coating with CaP. Elemental mapping images revealed that Zn, Ca, O and P elements were homogenously distributed on the ZnO_2_ surface (Fig. 1c), indicating the successful coating of CaP. These findings have been further confirmed by energy dispersive spectrometry (EDS) and X-ray photoelectron spectroscopy (XPS; Figs S4 and S5). In contrast to the absorption spectrum of Z@CaP, the absorption spectrum of Z@Ce6/CaP showed the characteristic absorption peaks of Ce6, indicating effective Ce6 loading during the coating process of CaP (Fig. S6). The N_2_ adsorption–desorption isotherm and corresponding pore diameter distribution curve showed that the pores on the Z@Ce6/CaP surface had an aperture of 3.8 nm and were conducive to the further loading of CPPO molecules (Fig. S7). The characteristic peaks of CPPO in the absorption spectrum of Z@Ce6/CaP@CB proved the successful loading of CPPO (Fig. S6). Finally, the surface of the nanocomposites was further modified with BSA to improve their biocompatibility, and the successful modification with BSA was proved by Fourier transform infrared spectroscopy (FT-IR; Fig. S8). The modification with BSA also resulted in a good dispersibility of Z@Ce6/CaP@CB in water, phosphate-buffered saline (PBS) and RPMI 1640 medium with an average hydrodynamic diameter of 121 nm (Figs S9 and S10).

In order to simulate the degradation properties of Z@Ce6/CaP@CB in acidic conditions, we measured the release of Ca^2+^ and Zn^2+^ under neutral (pH 7.4) and acidic (pH 4.5 and 6.5) conditions (Fig. 1d and e). Z@Ce6/CaP@CB only exhibited a significant release of Ca^2+^ and Zn^2+^ in an acidic environment, indicating effective degradation. The TEM image of Z@Ce6/CaP@CB after dispersion in an acidic environment for 24 h showed that the nanostructure disappeared completely, which further proves the acidic degradation properties of Z@Ce6/CaP@CB (Fig. 1f). Then, we assessed the H_2_O_2_ production ability of Z@CaP under acidic conditions. As shown in Fig. 1g, much more H_2_O_2_ (nearly 17%) was released from Z@CaP under acidic conditions than under neutral conditions after 2 h. All these results demonstrate that Z@Ce6/CaP@CB can be degraded under acidic conditions, resulting in the effective release of Ca^2+^, Zn^2+^ and H_2_O_2_. Furthermore, the ^1^O_2_ generation ability of Z@Ce6/CaP@CB was measured through the ROS sensor agent 1,3-diphenylisobenzofuran (DPBF) *in vitro*. As shown in Fig. 1h, compared with the control and Z@Ce6/CaP groups, the Z@Ce6/CaP@CB group showed significant DPBF consumption, indicating that the presence of CPPO and H_2_O_2_ enabled Ce6 to produce ^1^O_2_ without external energy stimulation. This is mainly because H_2_O_2_ released by ZnO_2_ under acidic conditions reacted further with CPPO to produce chemical energy to trigger the production of ^1^O_2_ by Ce6 (Fig. S11).

**Figure 2.**
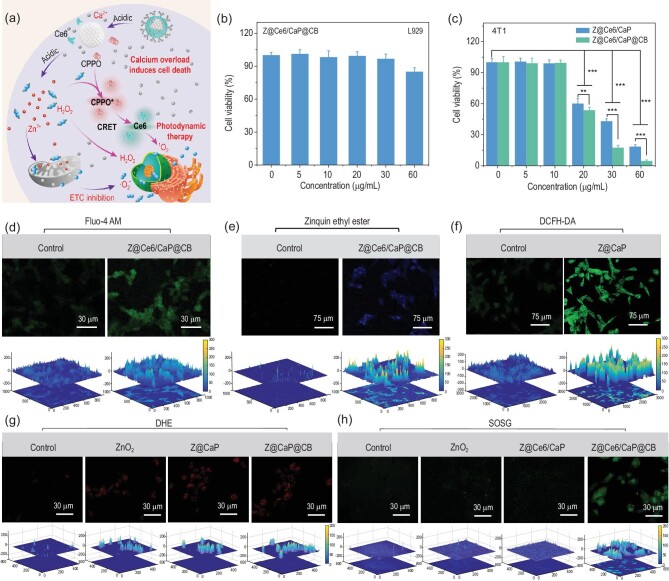
(a) Schematic illustration of the mechanism of Z@Ce6/CaP@CB-based synergistic therapy *in vitro*. (b) CCK-8 assay of L929 cells after treatment with different concentrations of Z@Ce6/CaP@CB. (c) CCK-8 assay of 4T1 cells after treatment with different concentrations of Z@Ce6/CaP and Z@Ce6/CaP@CB in medium. Detection of intracellular (d) Ca^2+^ and (e) Zn^2+^ contents by Fluo-4-AM probe and zinquin ethyl ester probe, respectively. (f) Intracellular H_2_O_2_ generation measured by DCFH-DA. (g) Intracellular ·O_2_^–^ generation measured by the DHE probe: (i) Control, (ii) ZnO_2_, (iii) Z@CaP and (iv) Z@CaP@CB. (h) ^1^O_2_ generation measured by the SOSG probe: (i) Control, (ii) ZnO_2_, (iii) Z@Ce6/CaP and (iv) Z@Ce6/CaP@CB. ^**^p < 0.01 and ^***^p < 0.001 by Student's two-tailed t test.

Inspired by the good ROS generation properties and on-demand Ca^2+^, Zn^2+^ and H_2_O_2_ release behaviors (Fig. 2a) of Z@Ce6/CaP@CB, we further evaluated its cytotoxicity and curative effect on mouse breast cancer cells (4T1) by the cell counting kit-8 (CCK-8) assay. Z@Ce6/CaP@CB did not show obvious cytotoxicity to normal cells (mouse fibroblast cells (L929)) after co-cultivation for 24 h (Fig. 2b), confirming the good biocompatibility of Z@Ce6/CaP@CB. In contrast, Z@Ce6/CaP@CB showed significant killing ability to cancer cells after co-culture with 4T1 cells for 24 h (Fig. 2c). Compared with the Z@Ce6/CaP group, the Z@Ce6/CaP@CB group showed a more significant growth inhibitory effect on cancer cells due to the generation of ^1^O_2_ by the CPPO-mediated chemiexcited photodynamic reaction. The cancer cell killing mechanism of Z@Ce6/CaP@CB was further investigated by fluorescence staining experiments. The intracellular Ca^2+^ and Zn^2+^ contents after Z@Ce6/CaP@CB treatment were evaluated using cell-permeable fluorescent Ca^2+^ probes (Fluo-4 AM) and Zn^2+^ probes (zinquin ethyl ester), as shown in Fig. 2d and e [18,23]. Compared with the control group, 4T1 cells showed significant enhancement of green and blue fluorescence after treatment with Z@Ce6/CaP@CB, indicating that Z@Ce6/CaP@CB was effectively endocytosed by 4T1 cells and further released Ca^2+^ and Zn^2+^. Furthermore, 2,7-dichlorofluorescin diacetate (DCFH-DA) fluorescence staining experiments showed strong green fluorescence of 4T1 cells after treatment with Z@CaP, indicating that ZnO_2_ effectively released H_2_O_2_ in the cells (Fig. 2f). In addition, Zn^2+^ released by ZnO_2_ increased the generation of mitochondrial ·O_2_^–^ and H_2_O_2_ by inhibiting the ETC, resulting in a rapid increase in endogenous ROS. As shown in Fig. 2g, we used dihydroethidium (DHE, ·O_2_^−^ probe) to verify the generation of endogenous ·O_2_^−^ caused by Zn^2+^ [26]. Compared with the control group, 4T1 cells showed strong enhancement of red fluorescence after treatment with ZnO_2_, Z@CaP and Z@CaP@CB, indicating that Zn^2+^ increased the content of endogenous ROS in the cells. Finally, the ^1^O_2_ generation capacity of Z@Ce6/CaP@CB was detected by singlet oxygen sensor green (SOSG, ^1^O_2_ probe) [27]. Compared with the control, ZnO_2_ and Z@Ce6/CaP groups, 4T1 cells showed strong enhancement of green fluorescence in the Z@Ce6/CaP@CB group, indicating that CPPO reacted with H_2_O_2_ produced by the degradation of ZnO_2_ to form a high-energy intermediate and consequently excited Ce6 to generate ^1^O_2_ (Fig. 2h). Under the oxidative stress caused by the generated ROS (H_2_O_2_, ^1^O_2_ and ·O_2_^–^), the ability of the cells to regulate Ca^2+^ declines gradually. Therefore, the large amount of Ca^2+^ released by Z@Ce6/CaP@CB in cells under oxidative stress can further trigger Ca^2+^ overload-induced cell death.

**Figure 3.**
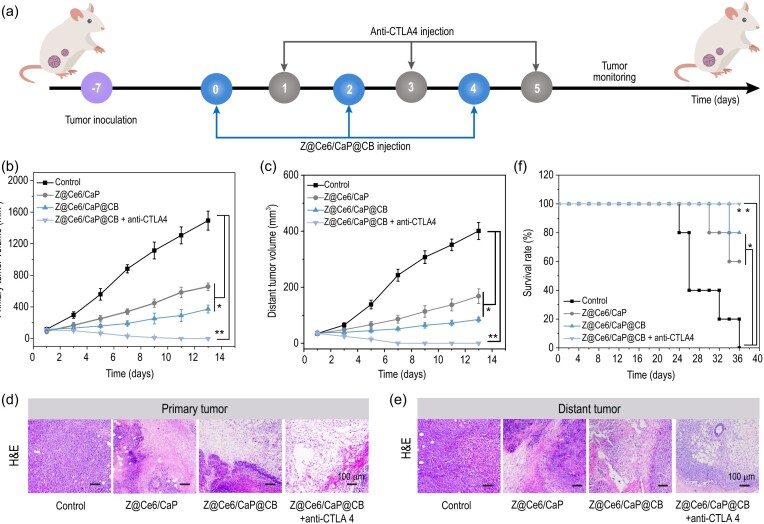
(a) Schematic illustration of the *in vivo* therapeutic mechanism. The tumor on the left side was used to evaluate the therapeutic effect of multiple ROS storms and Ca^2+^ overload, and the tumor on the right side was used to investigate the anti-tumor immunological effect activated during the treatment. Mice were injected intratumorally with Z@Ce6/CaP or Z@Ce6/CaP@CB on days 0, 2 and 4. Anti-CTLA4 was injected intravenously on days 1, 3 and 5. Volume curves of (b) primary tumor and (c) distant tumor: (i) Control, (ii) Z@Ce6/CaP, (iii) Z@Ce6/CaP@CB and (iv) Z@Ce6/CaP@CB + anti-CTLA4. H&E staining images of (d) primary tumor and (e) distant tumor slides from different treatment groups. (f) Survival percentages of the mice. ^*^p < 0.05 and ^**^p < 0.01 by Student's two-tailed t test.

Since Z@Ce6/CaP@CB shows good anti-cancer ability at the cellular level, its therapeutic effect on solid breast cancer tumors was further investigated *in vivo*. Based on the 4T1 tumor model, we found that the solid tumors of mice were significantly inhibited after the intravenous injection of nanodrugs (Fig. S12). Then, the double tumor model was used to verify whether Z@Ce6/CaP@CB produces a significant anti-tumor immune response during the treatment of primary tumors. The tumor on the left side was used to evaluate the therapeutic effect of multiple ROS storms and Ca^2+^ overload, while the tumor on the right side was used to investigate the anti-tumor immunological effect triggered during the treatment (Fig. 3a). Tumor-bearing mice were randomly divided into four groups (*n* = 5): (i) control group, (ii) Z@Ce6/CaP group, (iii) Z@Ce6/CaP@CB group and (iv) Z@Ce6/CaP@CB + anti-CTLA4 group. Compared with the control group, tumor-bearing mice treated with Z@Ce6/CaP or Z@Ce6/CaP@CB not only showed a good primary tumor growth inhibitory effect but also the growth of distant tumors without any treatment was significantly inhibited, indicating the activation of systemic anti-tumor immunity (Fig. 3b and c and Fig. S13). Although the activation of systemic anti-tumor immunity resulted in a good tumor growth inhibitory effect, the cytotoxic T-lymphocyte-associated protein 4 (CTLA4) immune checkpoint can prevent the activation and proliferation of T cells and seriously affect the effect of anti-tumor immunotherapy [28,29]. Therefore, to further enhance the anti-tumor effect of Z@Ce6/CaP@CB, we investigated the therapeutic effect of the combination of anti-CTLA4 and Z@Ce6/CaP@CB on tumors *in vivo*. As shown in Fig. 3b and c, primary and distant tumors in mice were completely suppressed after the combined treatment with anti-CTLA4 and Z@Ce6/CaP@CB, indicating that anti-CTLA4 effectively blocked CTLA4 and hindered the activity of immunosuppressive regulatory T cells (Tregs). Hematoxylin and eosin (H&E) staining and terminal deoxynucleotidyl transferase-mediated deoxyuridine triphosphate (dUTP) nick end labeling (TUNEL) staining assay further demonstrated that apoptosis of tumor cells was caused by a synergistic treatment with Z@Ce6/CaP@CB (Fig. 3d and e and Fig. S14). In addition, tumor-bearing mice also showed a significant survival rate after treatment with Z@Ce6/CaP@CB and anti-CTLA4 (Fig. 3f). Moreover, the weight of mice did not change significantly during the treatment period, indicating that the combined treatment with Z@Ce6/CaP@CB and anti-CTLA4 had no obvious side effects on the mice (Fig. S15). More importantly, Z@Ce6/CaP@CB was almost completely metabolized and cleared out of the mice 21 days after intravenous injection (Fig. S16), and it also did not show any obvious long-term toxic side effects on normal tissues (Fig. S17). The above results indicate that Z@Ce6/CaP@CB has good biocompatibility and application potential.

**Figure 4.**
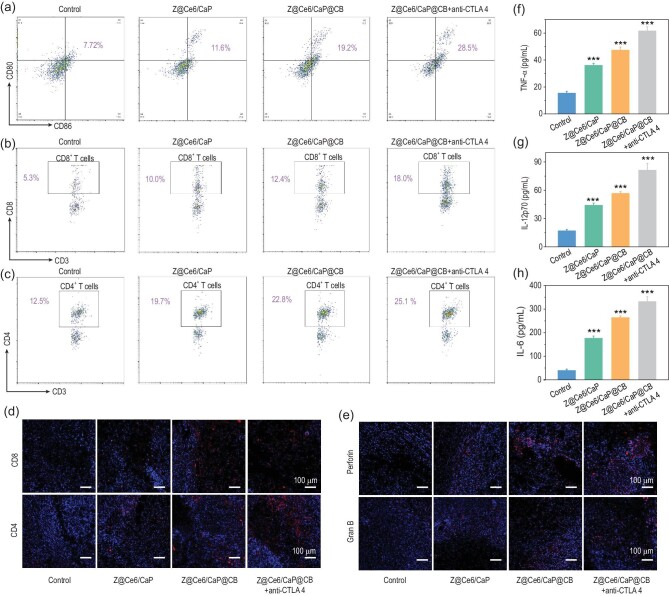
(a) CD80 and CD86 contents in lymph nodes of mice quantitatively detected by flow cytometry (gated on CD11c^+^ DC cells) on day 6 after different treatments: (i) Control, (ii) Z@Ce6/CaP, (iii) Z@Ce6/CaP@CB and (iv) Z@Ce6/CaP@CB + anti-CTLA4. Content of (b) CD4^+^ and (c) CD8^+^ T cells in splenocytes of mice (gated on CD3^+^ T cells) on day 6 after different treatments. (d) Immunofluorescence staining of distant tumor tissues of CD8 and CD4 cells on day 6 after different treatments. (e) Perforin and Gran B immunofluorescence staining of the distant tumor on day 6 after different treatments. (f–h) Secretion levels of pro-inflammatory cytokines (TNF-α, IL-6 and IL-12p70) in sera on day 6 after different treatments. ^***^p < 0.001 by Student's two-tailed t test.

In view of the significant distant tumor cure rate in the treated mice, the mechanism of the anti-tumor effect triggered by Z@Ce6/CaP@CB-based synergistic therapy in combination with anti-CTLA4 therapy was further studied. Dendritic cells (DCs) are the most powerful antigen-presenting cells (APCs), and can efficiently ingest, process and present antigens. Mature DCs can effectively activate initial T cells, which is a key role in initiating, regulating and activating the immune response [30,31]. As shown in Fig. 4a, compared with the control group, the maturity of DCs was significantly improved after treatment with Z@Ce6/CaP@CB and anti-CTLA4. As the activation of T lymphocytes is the core of the immune response, we further evaluated the activation degree of T cells in different treatment groups. Compared with the control group, the content of cytotoxic T lymphocytes (CD8^+^ T cells) and helper T lymphocytes (CD4^+^ T cells) in Z@Ce6/CaP, Z@Ce6/CaP@CB and Z@Ce6/CaP@CB + anti-CTLA4 groups was significantly increased, showing that CD8^+^ and CD4^+^ T cells were significantly activated under synergistic treatment with Z@Ce6/CaP@CB (Fig. 4b and c and Fig. S18). At the same time, the significant enhancement of the content of CD8^+^ and CD4^+^ T cells in distant tumors after treatment with Z@Ce6/CaP, Z@Ce6/CaP@CB and Z@Ce6/CaP@CB + anti-CTLA4 was confirmed by immunofluorescence staining experiments (Fig. 4d). Perforin and Granzyme B (Gran B) are important natural immune effectors in the body, mainly released by CD8^+^ T cells after contact with tumor cells [32,33]. As shown in Fig. 4e, perforin and Gran B were highly expressed in the Z@Ce6/CaP, Z@Ce6/CaP@CB and Z@Ce6/CaP@CB + anti-CTLA4 groups. In addition, the secretion of cytokines also plays an important role in anti-tumor immunity. Therefore, we further evaluated the secretion of pro-inflammatory cytokines (TNF-α, IL-6 and IL-12p70) in serum samples by enzyme linked immunosorbent assay (ELISA). All treatment groups exhibited higher secretion levels of pro-inflammatory factors than the control group (Fig. 4f–h), indicating that the combined effect of Z@Ce6/CaP@CB and anti-CTLA4 effectively activated the anti-tumor immunity.

**Figure 5.**
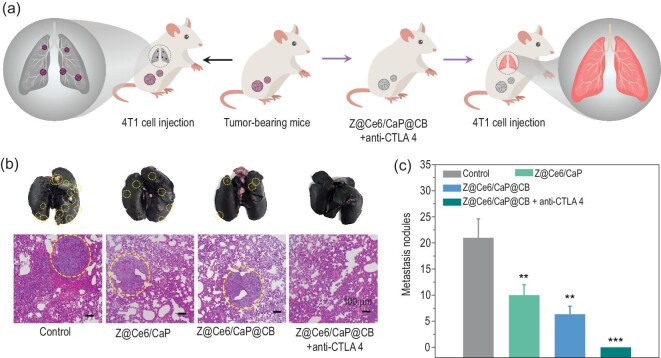
(a) Scheme of the synergistic effect of Z@Ce6/CaP@CB and anti-CTLA4 adjuvant immunotherapy on the inhibition of lung metastasis. (b) Representative photographs of lung metastasis nodules and H&E-stained images of lung tissues in different treatment groups: (i) Control, (ii) Z@Ce6/CaP, (iii) Z@Ce6/CaP@CB and (iv) Z@Ce6/CaP@CB + anti-CTLA4. (c) Calculated lung metastasis nodules of the mice after different treatments. ^**^p < 0.01 and ^***^p < 0.001 by Student's two-tailed t test.

We further verified the synergistic effect of Z@Ce6/CaP@CB and anti-CTLA4 adjuvant immunotherapy by evaluating tumor metastasis in the lungs (Fig. 5a). Compared with the control group, the number of metastatic nodules in the lungs of mice after treatment with Z@Ce6/CaP or Z@Ce6/CaP@CB was significantly reduced, and almost no cancer cell metastasis was found in the lungs due to the synergistic effect of Z@Ce6/CaP@CB and CTLA4 blocking adjuvant immunotherapy (Fig. 5b and c). H&E staining experiments showed that almost no tumor tissue was present in the lung tissue after treatment with Z@Ce6CaP@CB and CTLA4 (Fig. 5b). All the above results indicate that synergistic treatment with Z@Ce6/CaP@CB and anti-CTLA4 adjuvant immunotherapy can effectively inhibit the metastasis of malignant tumors by activating the body's anti-tumor immune response.

## CONCLUSION

In summary, we designed a cascaded ROS nanobomb (Z@Ce6/CaP@CB), which can effectively treat breast cancer tumors without external energy excitation. Z@Ce6/CaP@CB can effectively release Ca^2+^, Zn^2+^ and H_2_O_2_ through gradual degradation in the specific acidic TME. Released Zn^2+^ could increase the generation of mitochondrial ·O_2_^–^ and H_2_O_2_ by inhibiting the ETC, achieving a rapid increase in endogenous ROS. At the same time, the release of large amounts of H_2_O_2_ can cause oxidative damage to cancer cells and further activate CPPO-mediated chemiexcited PDT. In addition, the generated H_2_O_2_, ·O_2_^–^ and ^1^O_2_ can further trigger Ca^2+^ overload. Therefore, the introduction of Z@Ce6/CaP@CB nanobombs triggered a domino effect and effectively inhibited primary tumors through the synergistic effect of multiple ROS storms and Ca^2+^ overload, but also activated the systemic anti-tumor immune response to effectively inhibit distant tumors and metastases.

## METHODS

Preparation of ZnO_2_, Z@Ce6/CaP and Z@Ce6/CaP@CB; cytotoxicity test; detection of intracellular Ca^2+^ and Zn^2+^ concentrations; intracellular H_2_O_2_, ·O_2_^−^ and ^1^O_2_ production; *in vivo* anti-tumor effect; lung metastasis model; *ex vivo* analysis of different groups of immune cells; Gran B and perforin detection; and cytokine detection are shown in the Supplementary Data.

## Supplementary Material

nwab139_Supplemental_File

## References

[1] Yang B , Chen Y, Shi J et al. Reactive oxygen species (ROS)-based nanomedicine. Chem Rev 2019; 119: 4881–985. 10.1021/acs.chemrev.8b00626 30973011

[2] Lian M , Xue Z, Qiao X et al. Movable hollow nanoparticles as reactive oxygen scavengers. Chem 2019; 5: 2378–87. 10.1016/j.chempr.2019.05.023

[3] Perillo B , Donato MD, Pezone A et al. ROS in cancer therapy: the bright side of the moon. Exp Mol Med 2020; 52: 192–203. 10.1038/s12276-020-0384-2 32060354 PMC7062874

[4] Wei D , Yu Y, Zhang X et al. Breaking the intracellular redox balance with diselenium nanoparticles for maximizing chemotherapy efficacy on patient-derived xenograft models. ACS Nano 2020; 14: 16984–96. 10.1021/acsnano.0c06190 33283501

[5] Wu Z , Lim HK, Tan SJ et al Potent-by-design: amino acids mimicking porous nanotherapeutics with intrinsic anticancer targeting properties. Small 2020; 16: 2003757. 10.1002/smll.202003757 32686344

[6] Wu M , Ding Y, Li L. Recent progress in the augmentation of reactive species with nanoplatforms for cancer therapy. Nanoscale 2019; 11: 19658–83. 10.1039/C9NR06651A 31612164

[7] Ni K , Aung T, Li S et al. Nanoscale metal-organic framework mediates radical therapy to enhance cancer immunotherapy. Chem 2019; 5: 1892–913. 10.1016/j.chempr.2019.05.013 31384694 PMC6681452

[8] Zhang Z , Wang L, Liu W et al. Photogenerated-hole-induced rapid elimination of solid tumors by the supramolecular porphyrin photocatalyst. Natl Sci Rev 2021; 8: nwaa155. 10.1093/nsr/nwaa155 PMC8288340 34691632

[9] Yao C , Wang W, Wang P et al. Near-infrared upconversion mesoporous cerium oxide hollow biophotocatalyst for concurrent pH-/H_2_O_2_-responsive O_2_-evolving synergetic cancer therapy. Adv Mater 2018; 30: 1704833. 10.1002/adma.201704833 29315855

[10] Dai C , Zhang S, Liu Z et al. Two-dimensional graphene augments nanosonosensitized sonocatalytic tumor eradication. ACS Nano 2017; 11: 9467–80. 10.1021/acsnano.7b05215 28829584

[11] Li Z , Han J, Yu L et al. Synergistic sonodynamic/chemotherapeutic suppression of hepatocellular carcinoma by targeted biodegradable mesoporous nanosonosensitizers. Adv Funct Mater 2018; 28: 1800145. 10.1002/adfm.201800145

[12] Wang X , Zhang C, Du J et al. Enhanced generation of non-oxygen dependent free radicals by schottky-type heterostructures of Au−Bi_2_S_3_ nanoparticles via X-ray-induced catalytic reaction for radiosensitization. ACS Nano 2019; 13: 5947–58. 10.1021/acsnano.9b01818 30969747

[13] Guo Z , Zhu S, Yong Y et al. Synthesis of BSA-coated BiOI@Bi_2_S_3_ semiconductor heterojunction nanoparticles and their applications for radio/photodynamic/photothermal synergistic therapy of tumor. Adv Mater 2017; 29: 1704136. 10.1002/adma.201704136 29035426

[14] Tang Z , Zhang H, Liu Y et al. Antiferromagnetic pyrite as the tumor microenvironment-mediated nanoplatform for self-enhanced tumor imaging and therapy. Adv Mater 2017; 29: 1701683. 10.1002/adma.201701683 29094389

[15] Zhang C , Bu W, Ni D et al. Synthesis of iron nanometallic glasses and their application in cancer therapy by a localized Fenton reaction. Angew Chem Int Ed 2016; 55: 2101–6. 10.1002/anie.201510031 26836344

[16] Liu Y , Jiang Y, Zhang M et al. Modulating hypoxia via nanomaterials chemistry for efficient treatment of solid tumors. Acc Chem Res 2018; 51: 2502–11. 10.1021/acs.accounts.8b00214 30234960

[17] Liu Y , Zhen W, Wang Y et al. One dimensional Fe_2_P acts as a Fenton agent in response to NIR II light and ultrasound for deep tumor synergetic theranostics. Angew Chem Int Ed 2019; 58: 2407–12. 10.1002/anie.201813702 30600877

[18] Lin LS , Wang JF, Song JB et al. Cooperation of endogenous and exogenous reactive oxygen species induced by zinc peroxide nanoparticles to enhance oxidative stress-based cancer therapy. Theranostics 2019; 9: 7200–9. 10.7150/thno.39831 31695762 PMC6831298

[19] Indo HP , Davidson M, Yen HC et al. Evidence of ROS generation by mitochondria in cells with impaired electron transport chain and mitochondrial DNA damage. Mitochondrion 2007; 7: 106–18. 10.1016/j.mito.2006.11.026 17307400

[20] Jiang L , Bai H, Liu L et al. Luminescent, oxygen-supplying, hemoglobin-linked conjugated polymer nanoparticles for photodynamic therapy. Angew Chem Int Ed 2019; 58: 10660–5. 10.1002/anie.201905884 31173456

[21] Yu Z , Zhou P, Pan W et al. A biomimetic nanoreactor for synergistic chemiexcited photodynamic therapy and starvation therapy against tumor metastasis. Nat Commun 2018; 9: 5044. 10.1038/s41467-018-07197-8 30487569 PMC6262009

[22] Guan Q , Zhou LL, Lv FH et al. A glycosylated covalent organic framework equipped with BODIPY and CaCO_3_ for synergistic tumor therapy. Angew Chem Int Ed 2020; 59: 18042–7. 10.1002/anie.202008055 32589819

[23] Zhang M , Song R, Liu Y et al. Calcium-overload-mediated tumor therapy by calcium peroxide nanoparticles. Chem 2019; 5: 2171–82. 10.1016/j.chempr.2019.06.003

[24] Elbahri M , Abdelaziz R, Disci-Zayed D et al. Underwater Leidenfrost nanochemistry for creation of size-tailored zinc peroxide cancer nanotherapeutics. Nat Commun 2017; 8: 15319. 10.1038/ncomms15319 28497789 PMC5437293

[25] Liu S , Li W, Dong S et al. Degradable calcium phosphate-coated upconversion nanoparticles for highly efficient chemo-photodynamic therapy. ACS Appl Mater Interfaces 2019; 11: 47659–70. 10.1021/acsami.9b11973 31713407

[26] Zhen W , Liu Y, Wang W et al. Specific ‘unlocking’ of a nanozyme-based butterfly effect to break the evolutionary fitness of chaotic tumors. Angew Chem Int Ed 2020; 59: 9491–7. 10.1002/anie.201916142 32100926

[27] Wang C , Cao F, Ruan Y et al. Specific generation of singlet oxygen via Russell mechanism in hypoxic tumor and GSH depletion by Cu-TCPP nanosheets for cancer therapy. Angew Chem Int Ed 2019; 58: 9846–50. 10.1002/anie.201903981 31077533

[28] Xu J , Xu L, Wang C et al. Near-infrared-triggered photodynamic therapy with multitasking upconversion nanoparticles in combination with checkpoint blockade for immunotherapy of colorectal cancer. ACS Nano 2017; 11: 4463–74. 10.1021/acsnano.7b00715 28362496

[29] Chen Q , Chen J, Yang Z et al. Nanoparticle-enhanced radiotherapy to trigger robust cancer immunotherapy. Adv Mater 2019; 31: 1802228. 10.1002/adma.201802228 30663118

[30] Chang M , Wang M, Wang M et al. A multifunctional cascade bioreactor based on hollow structured Cu_2_MoS_4_ for synergetic cancer chemo-dynamic therapy/starvation therapy/phototherapy/immunotherapy with remarkably enhanced efficacy. Adv Mater 2019; 31: 1905271. 10.1002/adma.201905271 31680346

[31] Chang M , Hou Z, Wang M et al. Cu_2_MoS_4_/Au heterostructures with enhanced catalase-like activity and photoconversion efficiency for primary/metastatic tumors eradication by phototherapy-induced immunotherapy. Small 2020; 16: 1907146. 10.1002/smll.201907146 32162784

[32] Lu J , Liu X, Liao YP et al. Breast cancer chemo-immunotherapy through liposomal delivery of an immunogenic cell death stimulus plus interference in the IDO-1 pathway. ACS Nano 2018; 12: 11041–61. 10.1021/acsnano.8b05189 30481959 PMC6262474

[33] He S , Li J, Lyu Y et al. Near-infrared fluorescent macromolecular reporters for real-time imaging and urinalysis of cancer immunotherapy. J Am Chem Soc 2020; 142: 7075–82. 10.1021/jacs.0c00659 32196318

