# Supplementary material for: Cascade-responsive nanobomb with domino effect for anti-tumor synergistic therapies
Source: Natl Sci Rev. 2021 Aug 9;9(3):nwab139. doi: 10.1093/nsr/nwab139 (PMC8970328; doi:10.1093/nsr/nwab139)
Supplement: nwab139_Supplemental_File [file nwab139_supplemental_file.doc]

Supporting Information

Cascade-Responsive Nanobomb with Domino Effect for Anti-Tumor Synergistic Therapies

Yang Liu, Yinghui Wang*, Shuyan Song, and Hongjie Zhang*

Y. Liu, Dr. Y. H. Wang, Prof. S. Y. Song, Prof. H. J. Zhang

State Key Laboratory of Rare Earth Resource Utilization, Changchun Institute of Applied Chemistry, Chinese Academy of Sciences, Changchun, Jilin, 130022, P. R. China

Y. Liu, Prof. S. Y. Song, Prof. H. J. Zhang

School of Applied Chemistry and Engineering, University of Science and Technology of China, Hefei, Anhui 230026, P. R. China

Prof. H. J. Zhang

Department of Chemistry, Tsinghua University, Beijing 100084, PR China

E-mail: [yhwang@ciac.ac.cn,](mailto:yhwang@ciac.ac.cn,) hongjie@ciac.ac.cn,

Experimental Section

**Preparation of ZnO_2_:** ZnO_2_ were synthesized according to a modified method reported previously. In detail, 0.46 g of zinc acetate (Acros) was dissolved in 49 mL of deionized water. Then, 1mL of hydrogen peroxide (Beijing Chemical Reagents Company) was added to zinc acetate solution. Then, the mixture was suddenly introduced into a superheated plate at a temperature of 300℃. Finally, the ZnO_2_ precipitate was obtained by centrifugation at 12000 rpm for 5 minutes. The products were washed several times with deionized water.

**Preparation of Z@Ce6/CaP:** The coating method of CaP was accorded to a modified method reported previously. 1.5 mg of Ca(OH)_2_ (Aladdin) and 4 mg of PVP (Aladdin) were dispersed in 10 mL of distilled water to form a transparent solution. Then, 4 mg/mL of ZnO_2_ solution was added to the mixture. The mixture was sonicated for 30 min, and then 20 mL of isopropyl alcohol was added into the above solution drop by drop under violent stirring. Finally, 100 μL of Na_2_HPO_4_ (50 mg/mL, Beijing Chemical Reagents Company) was added dropwise into the solution and the mixture was stirred for 12 h. The effective load of Ce6 can be realized by adding Ce6 (FrontierSci) during the this process. The Z@Ce6/CaP precipitate was obtained by centrifugation.

**Preparation of Z@Ce6/CaP@CB:** 6 mg of Z@Ce6/CaP was added into 1 mL of THF solution containing 3 mg of CPPO (Macklin). After the mixture was sonicated for 2 min, THF was removed by vacuum to obtain Z@Ce6/CaP@C. Finally, the Z@Ce6/CaP@C solution was added dropwise into the BSA solution and stirred for 12 h. The Z@Ce6/CaP@CB was obtained by centrifugation.

**Cytotoxicity test:** 4T1 Cells were inoculated into 96-well plates for 24 h. Then, different concentrations of Z@Ce6/CaP and Z@Ce6/CaP@CB medium solution (Z@Ce6/CaP: 0, 5, 10, 20, 30, and 60 μg/mL) were added. After 24 h, 4T1 cells were washed three times with PBS, and the activity of cells was tested by Cell Counting Kit-8 assay kit (Bioss).

**Detection of intracellular Ca^2+^ and Zn^2+^ concentration:** 4T1 Cells were inoculated into glass bottom culture dishes for 24 h. Then, adding Z@Ce6/CaP@CB medium solution (Z@Ce6/CaP: 60 μg/mL) to continue co-culture for 4 h. The treated 4T1 cells were further incubated with cell-permeable fluorescent Ca^2+^ probe Fluo-4 AM (4 μM, Beyotime). The fluorescence signal was measured by CLSM. To detect intracellular Zn^2+^ concentration, cell-permeable fluorescent Zn^2+^ probe (zinquin ethyl ester, 25 μM, MKBio) was applied in accordance with the above steps.

**Intracellular H_2_O_2_,**·**O_2_^−^, and ^1^O_2_ production:** 4T1 Cells were inoculated into glass bottom culture dishes for 24 h. Then, the 4T1 cells were treated with different conditions. The treated 4T1 cells were further incubated with DCFH-DA (10 μM, Sigma-Aldrich) to detect the H_2_O_2_ production. Similarly, dihydroethidium (DHE) probe (30 μM, Beyotime) and singlet oxygen sensor green (SOSG, 10 μM, Thermo fisher) were applied in accordance with the above steps to detect the generation of ·O_2_^−^ and ^1^O_2_, respectively.

**In vivo anti-tumor effect:** All of the animal experiments were conducted according to the rules of the Institutional Animal Care and Use Committee of Jilin University (IACUC). 4T1 tumor cells were inoculated subcutaneously on the left and right sides of Balb/c mice. The tumor on the left was used to evaluate the synergistic therapeutic effect of multiple ROS storms, Ca^2+^ overload, and Zn-mediated ETC inhibition, while the tumor on the right was used to evaluate the anti-tumor immunological effect activated during the treatment. Tumor-bearing mice were randomly divided into four groups (n=5): (A) Control group, (B) Z@Ce6/CaP group, (C) Z@Ce6/CaP@CB group, and (D) Z@Ce6/CaP@CB + anti-CTLA4 group. Mice were injected intratumorally with Z@Ce6/CaP (3 mg/kg) or Z@Ce6/CaP@CB (Z@Ce6/CaP: 3 mg/kg) on days 0, 2, and 4 d. The the anti-CTLA4 (1 mg/kg) was injected intravenously on days 1, 3, and 5 d. The body weight and tumor volume of mice were recorded every two days. Tumor sections of each group were further studied by hematoxylin and eosin (H&E) and terminal deoxynucleotidyl transferase mediated dUTP nick end labeling (TUNEL) staining assay.

**Lung metastasis model**: The untreated Balb/c mice and Z@Ce6/CaP, Z@Ce6/CaP@CB, and Z@Ce6/CaP@CB + anti-CTLA4 cured mice were intravenously injected 4T1 cells. After 30 d, lungs were stained by India ink for metastatic nodule counting. In addition, lungs sections were harvested from each group and further studied by H&E staining assay.

**Ex vivo analysis of different groups of immune cells.** To examine the immune response caused by the combinational therapy, lymph nodes, distant tumors and spleens of mice were harvested. The lymph nodes, distant tumors and spleens were made into a single cell suspension according to the specified procedure. The collected lymphocytes were incubated with anti-CD11c-PerCP/Cyanine5.5 (0.2 mg/mL, ≤ 1.0 µg per million cells in 100 µl volume), anti-CD80-PE (0.2 mg/mL, ≤ 0.5 µg per million cells in 100 µl volume), and anti-CD86-APC (0.2 mg/mL, ≤ 0.25 µg per million cells in 100 µl volume) (BioLegend) to determine the maturity of DC cells. After incubated with anti-CD3-FITC (0.5 mg/mL, ≤ 1.0 μg per million cells in 100 μL volume), anti-CD8a-PE (0.2 mg/mL, ≤ 0.25 μg per million cells in 100 μL volume), anti-CD4-APC (0.2 mg/mL, ≤ 0.25 μg per million cells in 100 μL volume) (BioLegend), the content of CD8^+^ and CD4^+^ T cells were detected by flow cytometry analysis (Guava EasyCyte). The content of CD8^+^ and CD4^+^ T cells in distant tumor tissues were further evaluated through immunofluorescence staining (anti-CD8 (10 μg/ml) and anti-CD4 (10 μg/ml, Bioss))

**Granzyme B and perforin detection:** Immunofluorescence staining in tumor tissues for granzyme B and perforin of different treatment groups were evaluated through staining with anti-Granzyme B (10 μg/ml) and anti-Perforin (10 μg/ml, Bioss).

**Cytokine detection:** Serum samples were isolated from mice after different treatments and diluted for analysis. TNF-α, IL-12p70, and IL-6 were measured by ELISA Kit (DAKEWE).

**Results and Discussion**


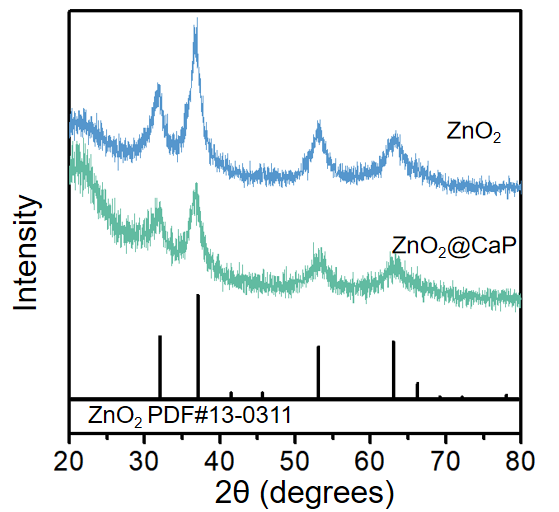


**Figure S1**. XRD of ZnO_2_ and ZnO_2_@CaP.

No discernible peaks of crystalline CaP were observed in the XRD pattern. However, compared with the XRD of ZnO_2_, the broad peak occurred at around 2θ=30° in XRD of Z@Ce6/CaP, indicating anamorphous phase of CaP was formed.


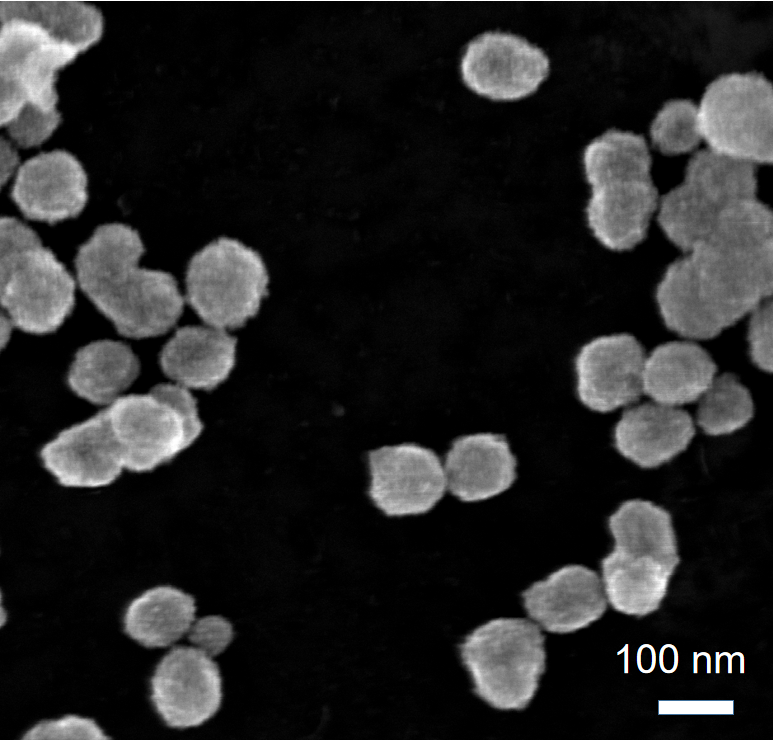


**Figure S2**. SEM of Z@Ce6/CaP@CB.


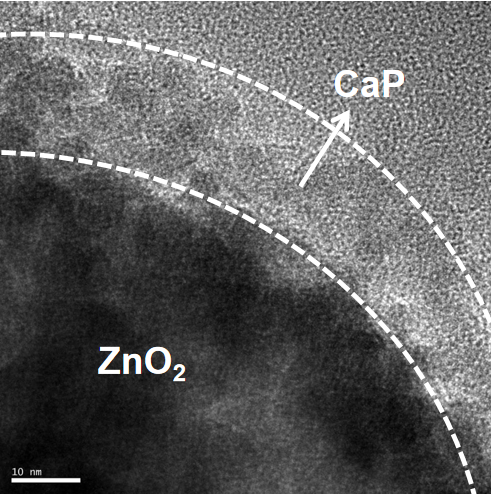


**Figure S3.** Magnified TEM image of Z@Ce6/CaP@CB.


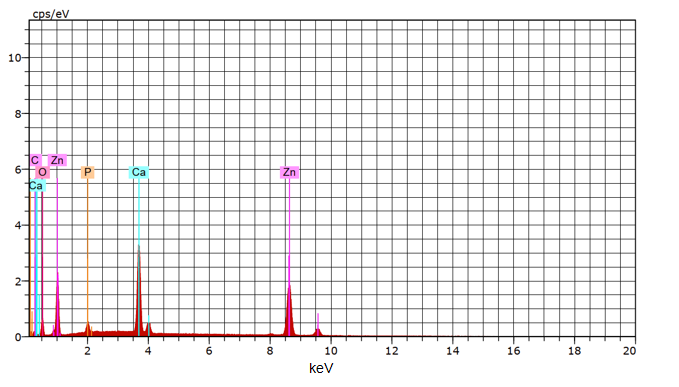
**Figure S4**. EDX of Z@CaP.


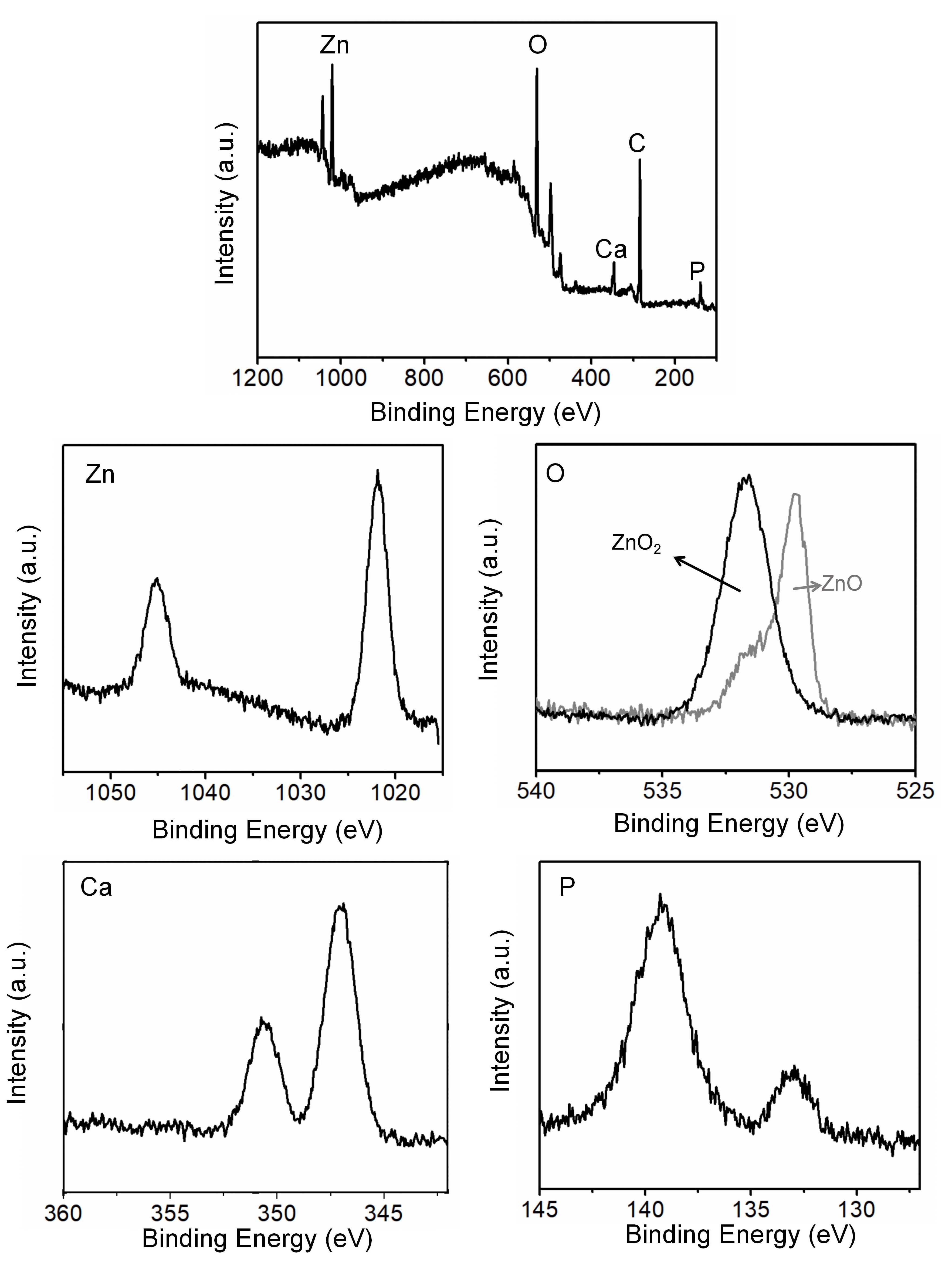


**Figure S5**. XPS spectra of Z@CaP, Zn, O, Ca, and P.


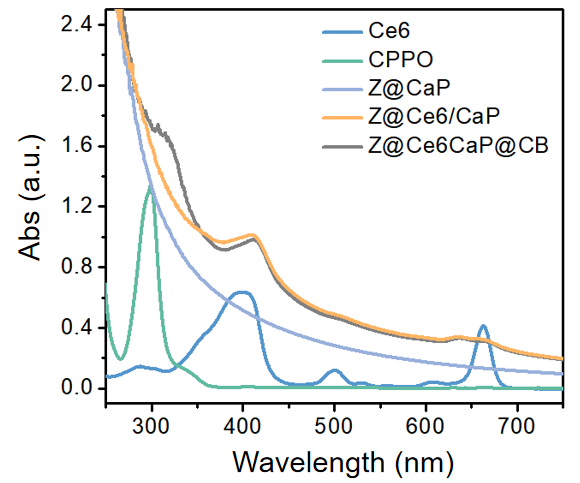


**Figure S6**. UV-vis absorption spectra of Ce6, CPPO, Z@CaP, Z@Ce6/CaP, and Z@Ce6/CaP@CB.


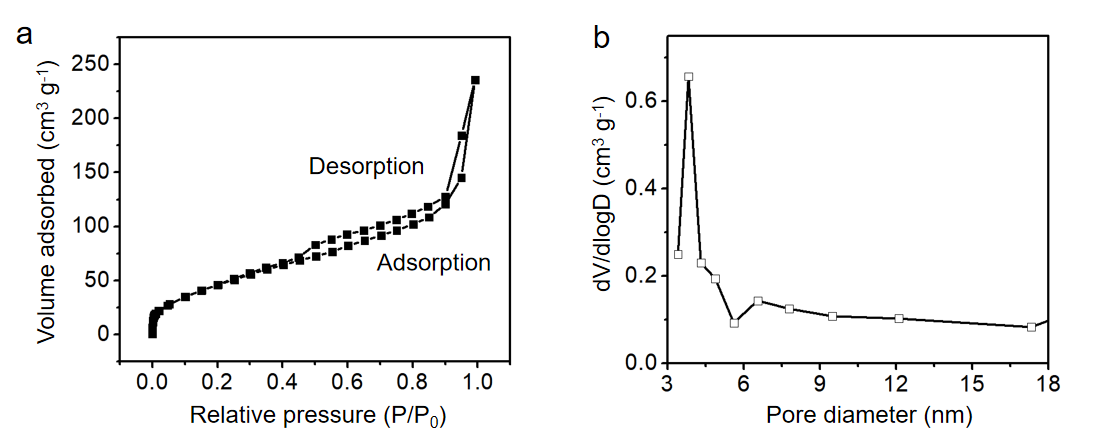


**Figure S7**. (a) N_2_ adsorption-desorption isotherm and (b) pore size distribution of Z@Ce6/CaP.

.
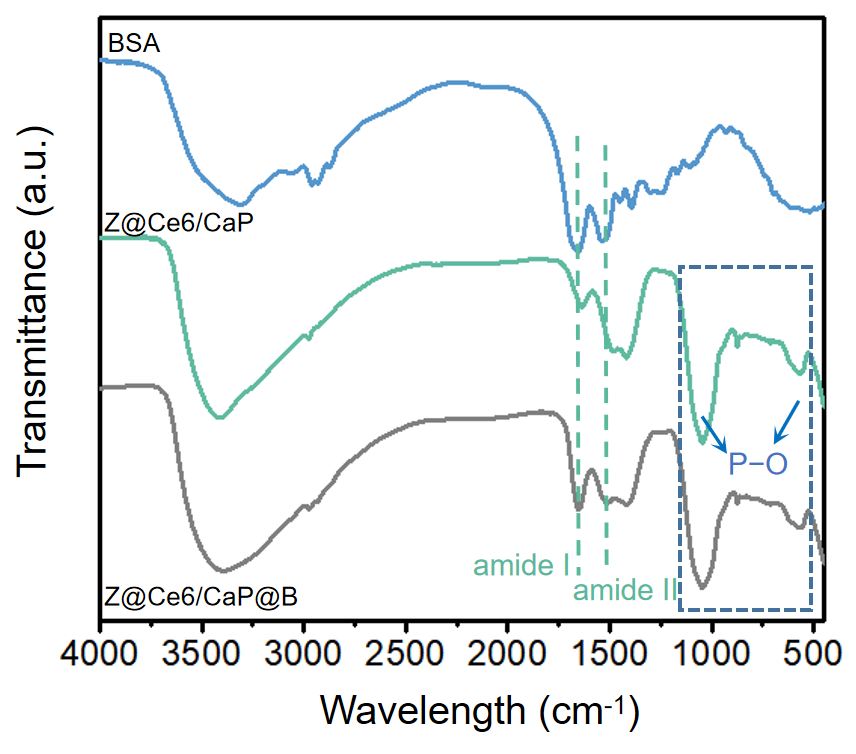


**Figure S8**. FT-IR spectra of BSA, Z@Ce6/CaP, and Z@Ce6/CaP@B.


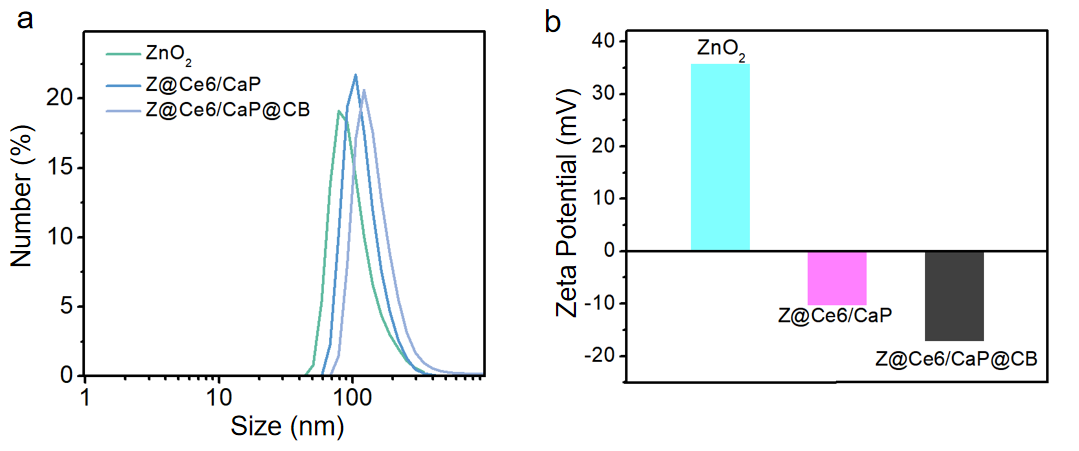


**Figure S9**. (a) Hydrodynamic size and (b) Zeta potential of ZnO_2_, Z@Ce6/CaP, and Z@Ce6/CaP@CB in H_2_O.

The hydrodynamic size of the different nanoparticles was measured using dynamic light scattering, the diameter increased continuously with each step of the process. Moreover, the zeta potential values provided further evidence for the successful construction of nanoparticles in each procedure.


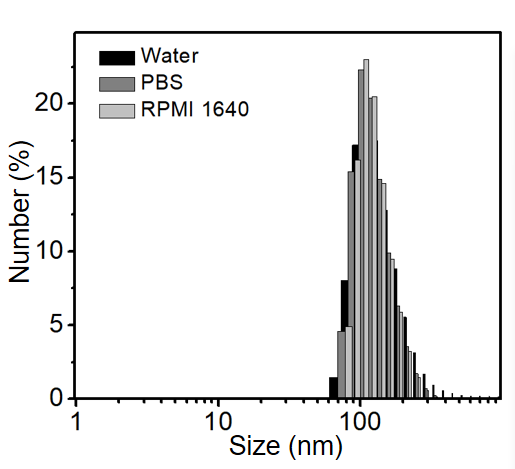


**Figure S10**. Hydrodynamic size of Z@Ce6/CaP@CB in H_2_O, PBS, and RPMI 1640.


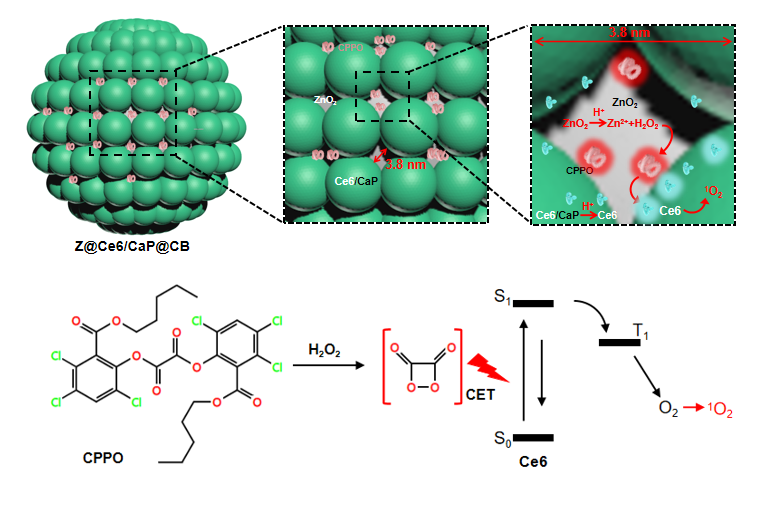


**Figure S11**. Mechanism of the chemiexcited production of ^1^O_2_.

Chemiluminescence resonance energy transfer (CRET) refers to a nonradiative transfer mechanism between chemiluminescence donors and adjacent acceptors, usually located within 10 nm. As shown in Figure S7, Z@Ce6/CaP have pores with 3.8 nm aperture on the surfaces, CPPO is mainly loaded in these pores and has a short distance from Z@Ce6/CaP. Moreover, the degradation of ZnO_2_ and CaP occurs at the same time, H_2_O_2_ is already produced at the beginning of the degradation. Hence, the distance between H_2_O_2_, CPPO, and Ce6 in pores with 3.8 nm aperture is very close, and they have sufficient contact probability with each other. Therefore, although Z@Ce6/CaP@CB can be degraded, it still can produce ^1^O_2_ through CRET.


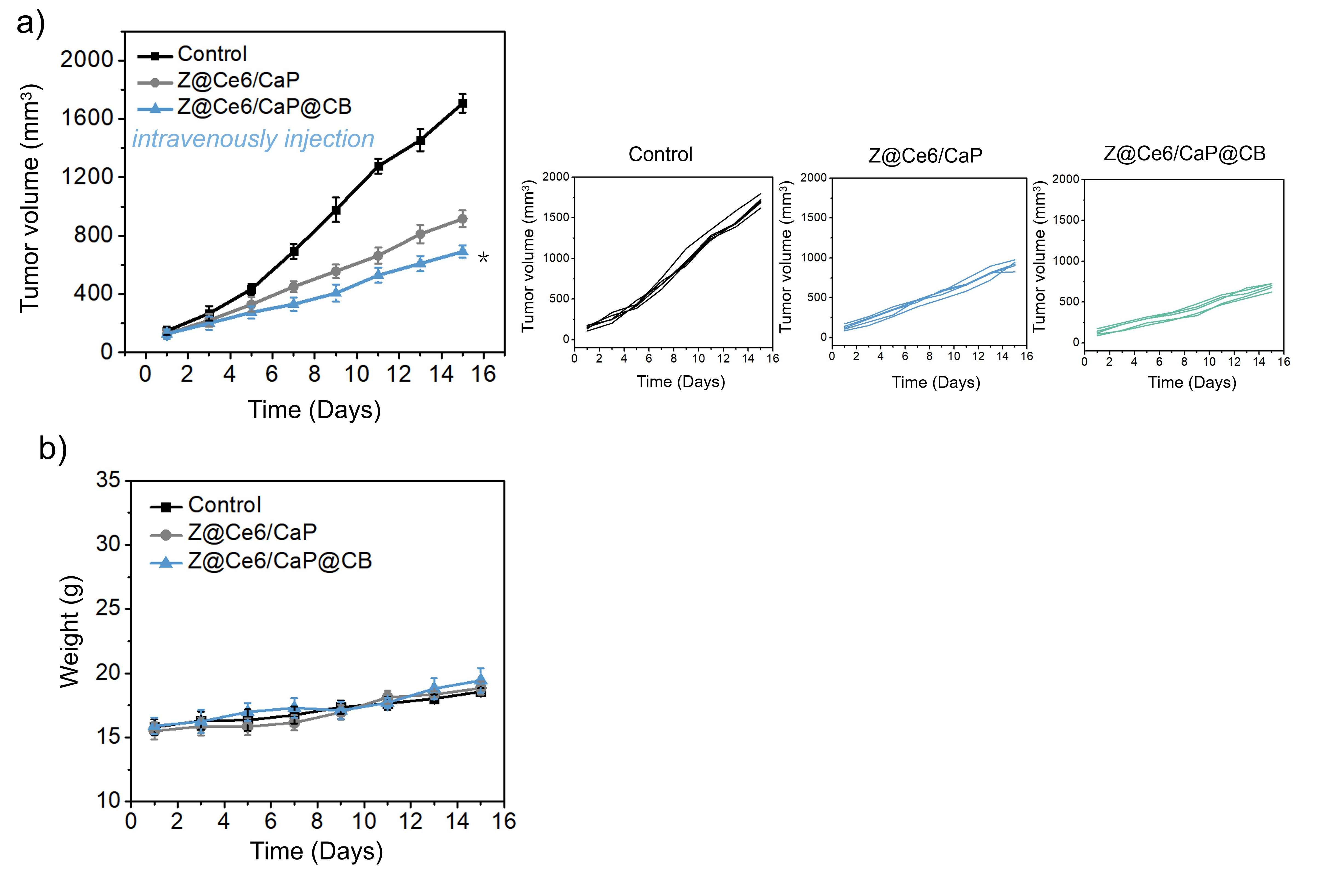


**Figure S12.** (a) Volume curves of tumor (n=5): (1) Control, (2) Z@Ce6/CaP, and (3) Z@Ce6/CaP@CB. During treatment, mice were injected intravenously with Z@Ce6/CaP (3 mg/kg) or Z@Ce6/CaP@CB (Z@Ce6/CaP: 3 mg/kg). The tumor sizes were measured every 2 days. (b) Body weights of mice after different treatments. *p < 0.05 by student’s two-tailed t test.


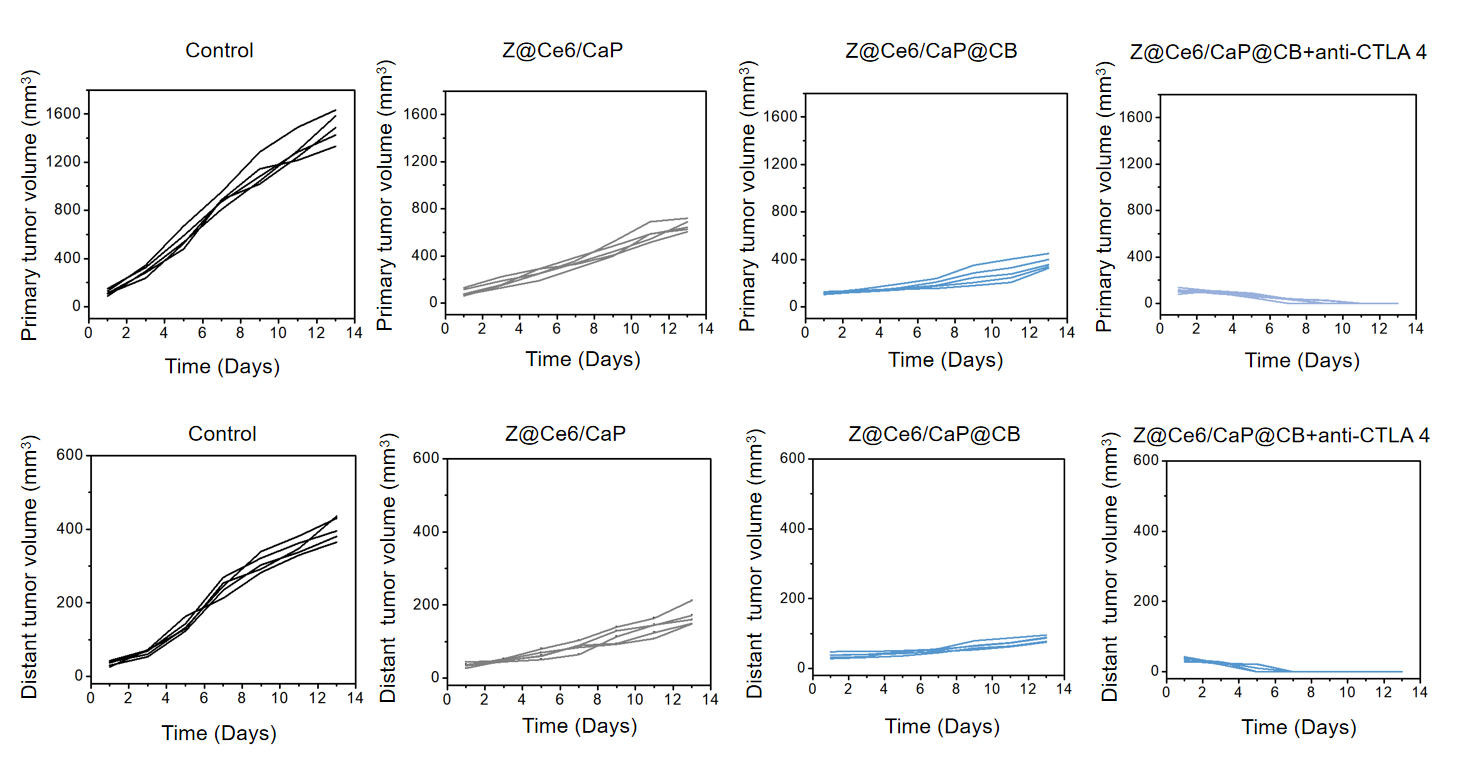


**Figure S13.** Volume curves of primary and distant tumor: (1) Control, (2) Z@Ce6/CaP, (3) Z@Ce6/CaP@CB, and (4) Z@Ce6/CaP@CB + anti-CTLA4.


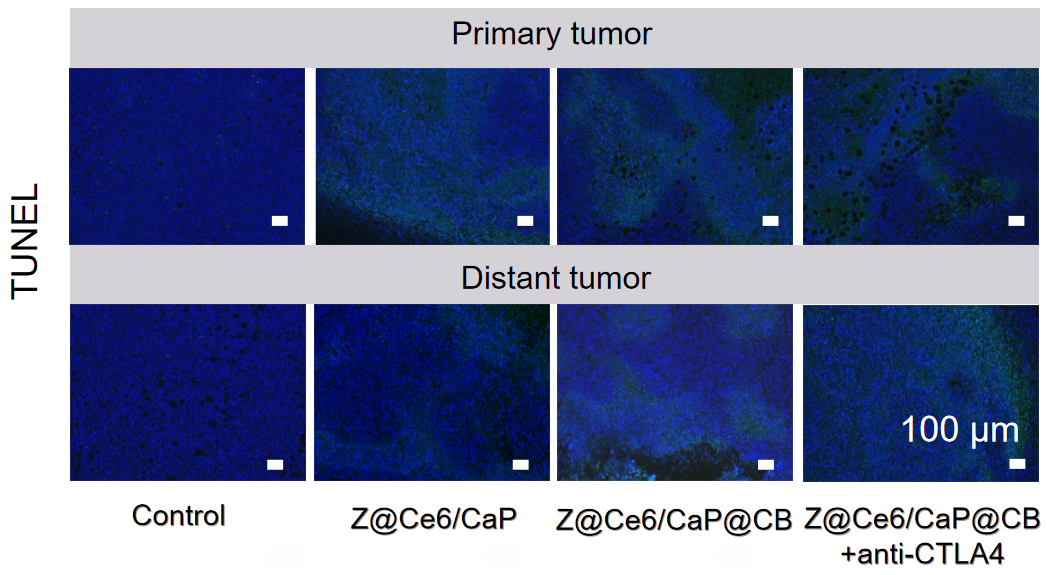


**Figure S14.** TUNEL staining images of primary tumor and distant tumor slides from different treatment groups.


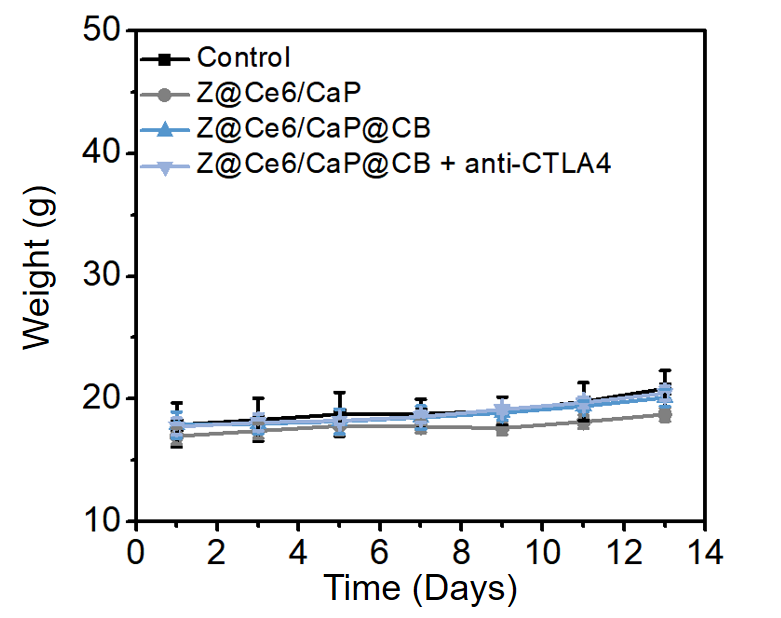


**Figure S15.** Body weights of mice after different treatments.


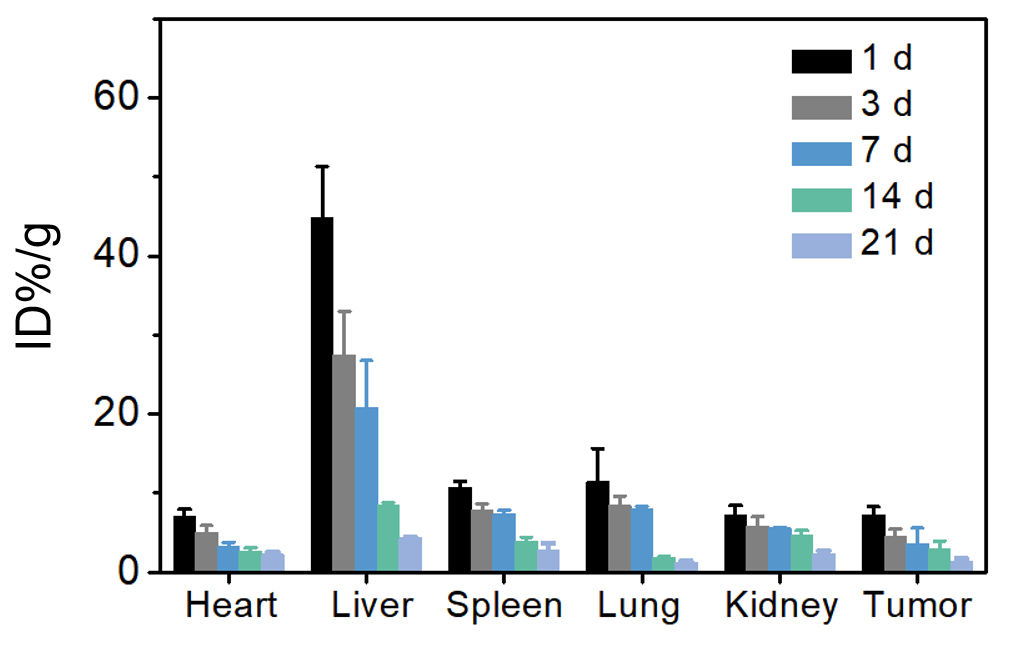


**Figure S16.** Biodistribution of Zn in the tumor and main organs at different times. Z@Ce6/CaP@CB could accumulate in tumor sites through the enhanced permeability and retention (EPR) effect.


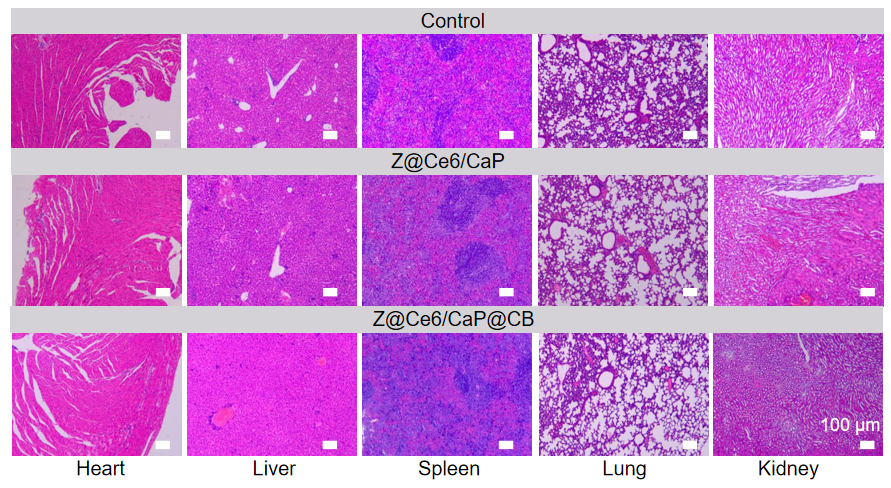


**Figure S17.** H&E staining of major organs slides.


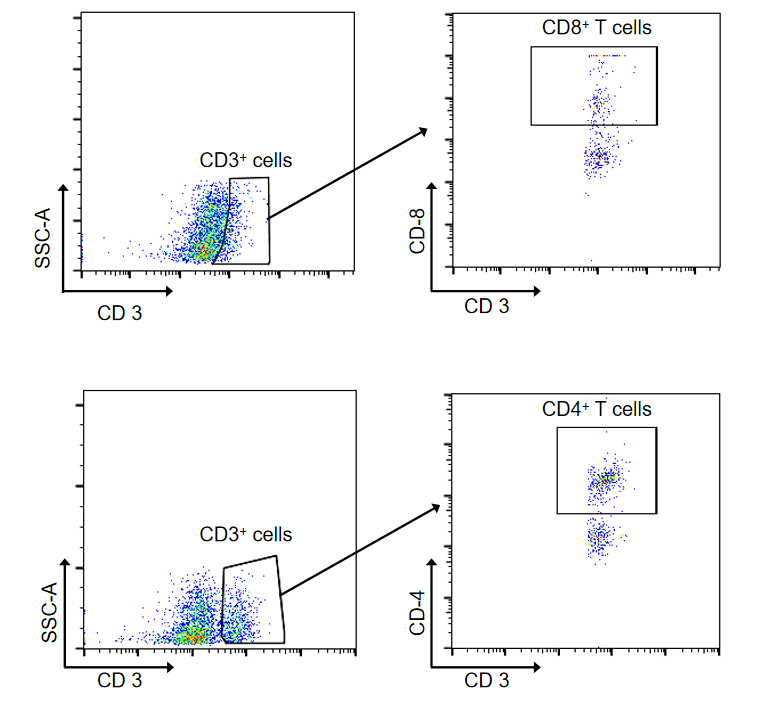


**Figure S18.** Gating strategies for CD8^+^ and CD4^+^ T cells.
